# Tetanus Following the Hypodermic Injection of Quinine

**Published:** 1876-08

**Authors:** 


Tetanus Following the Hypodermic Injection of Quinine. Dr. Roberts, surgeon of the 9th Regiment of Sepoy Infantry, reports two fatal cases of tetanus following the hypodermic injection of quinine for malarial fever, both occurring in the same week. The first patient received an injection on October 15th, and one on the 16th, each containing three and a half grains of the neutral sulphate of quinine. The chills were arrested and the patient returned to duty six days later, but on the same evening he was taken with tetanic spasms, and died on October 25th. The second case received one injection of quinine on October 17th, which stopped the chills. He was discharged from hospital on the 24th, but on the morning of the 25th he complained of stiffness in the jaws and neck, and died at noon on the next day. These cases were treated with chloral, by the mouth and by hypodermic injection, morphia, and chloroform. In neither case was there a trace of a lesion of a nerve at the site of the original injections. The doctor attributed the first case to rapid changes in the temperature, cases of idiopathic tetanus being not uncommon in some parts of India at the commencement of the rainy season; but with the second case the coincidence became so marked that he was obliged to admit the connection between the tetanus and the hypodermic injections. There was no evidence in either case that poison had been administered. The doctor had given quinine hypodermically some hundreds of times previously; had always taken it in that way himself, and the civil surgeon of the post had used it upwards of five thousand times, and in all these cases there had not been an authenticated instance of sloughing even, following this treatment. Why, then, should it produce tetanus in these two cases?—*The Lancet*, May 20th.

The "Florida Cough."—The *New York Gazette* has the following hit: The most popular fashionable affectation among young ladies ravenous for social notoriety is the "Florida cough," which is regarded by those who have been abroad as a fine substitute for "Roman malaria," so fashionable a few years ago. The Southern malady is supposed to be contracted sitting on the piazza of a Magnolia or Jacksonville hotel, flirting and eating oranges alternately. Those who have never been near either place suffer dreadfully from the disease.

